# Conformational heterogeneity and bubble dynamics in single bacterial transcription initiation complexes

**DOI:** 10.1093/nar/gkx1146

**Published:** 2017-11-21

**Authors:** Diego Duchi, Kristofer Gryte, Nicole C Robb, Zakia Morichaud, Carol Sheppard, Konstantin Brodolin, Sivaramesh Wigneshweraraj, Achillefs N Kapanidis

**Affiliations:** 1 Gene Machines Group, Biological Physics Research Unit, Clarendon Laboratory, Department of Physics, University of Oxford, Oxford OX1 3PU, UK; 2 CNRS UMR 9004, Institut de Recherche en Infectiologie de Montpellier, Université de Montpellier, 1919 route de Mende, 34293 Montpellier, France; 3 MRC Centre for Molecular Microbiology and Infection, Imperial College London, London SW7 2AZ, UK

## Abstract

Transcription initiation is a major step in gene regulation for all organisms. In bacteria, the promoter DNA is first recognized by RNA polymerase (RNAP) to yield an initial closed complex. This complex subsequently undergoes conformational changes resulting in DNA strand separation to form a transcription bubble and an RNAP-promoter open complex; however, the series and sequence of conformational changes, and the factors that influence them are unclear. To address the conformational landscape and transitions in transcription initiation, we applied single-molecule Förster resonance energy transfer (smFRET) on immobilized *Escherichia coli* transcription open complexes. Our results revealed the existence of two stable states within RNAP–DNA complexes in which the promoter DNA appears to adopt closed and partially open conformations, and we observed large-scale transitions in which the transcription bubble fluctuated between open and closed states; these transitions, which occur roughly on the 0.1 s timescale, are distinct from the millisecond-timescale dynamics previously observed within diffusing open complexes. Mutational studies indicated that the σ^70^ region 3.2 of the RNAP significantly affected the bubble dynamics. Our results have implications for many steps of transcription initiation, and support a bend-load-open model for the sequence of transitions leading to bubble opening during open complex formation.

## INTRODUCTION

Transcription initiation is the most highly regulated step in gene expression. In bacteria, the protein responsible for transcription initiation is RNA polymerase (RNAP), a multi-subunit enzyme consisting of a catalytic core (α_2_ββ’ω) and an initiation sigma (σ) factor. The most abundant σ factor in *Escherichia coli* is σ^70^, which uses its conserved regions 2 and 4 to enable RNAP to recognize the −10 and −35 promoter elements respectively (1). Initial promoter DNA binding yields the RNAP–promoter closed complex (RP_C_), which subsequently undergoes large conformational changes that trigger promoter DNA strand separation over 12–14 bp, resulting in the formation of the catalytically competent RNAP–promoter open complex (RP_O_) (2–4). The formation of the open complex is one of the most complex and least understood transitions in the transcription initiation of all organisms, including bacteria.

A model of bacterial RP_O_ formation has emerged through structural, chemical footprinting, and rapid mixing kinetic approaches (5–10). The model posits three keys aspects of transcription initiation. First, RP_O_ formation occurs via multiple isomerization steps, which involve structurally distinct intermediates (7,11,12). Second, bubble melting starts at the −10 element before propagating downstream to form the fully open transcription bubble (13,14). Third, different RNAP structural regions play distinct roles during transcription initiation (2); σ^70^ region 2.3 plays a central role in transcription bubble nucleation (15); σ^70^ region 3.2 and the β’ switch-2 elements correctly position the melted template (T) strand at the active site (15–18); and finally, the core RNAP downstream jaw and clamp bind to downstream DNA and stabilize RP_O_ (4,6,19).

The stability of RP_O_ complexes depends substantially on the promoter sequences involved. For example, promoters such as *lac*UV5 and λP_R_ form very stable RP_O_ (lifetimes of 1 h for *lac*UV5 (20) and 11 h for λP_R_ at 37°C (21)), whereas the RP_O_ formed on ribosomal RNA promoters (such as *rrn*B) is very unstable (lifetime of 1 min; (22)). However, despite the fact that RP_O_ can be a very stable complex, recent evidence points to the presence of significant dynamics within RP_O_. Specifically, our previous single-molecule FRET (smFRET) studies on diffusing transcription complexes using a variant of the *lac*UV5 promoter showed that the template DNA within RP_O_ exhibits dynamic heterogeneity (23). Furthermore, smFRET studies on surface-immobilized mitochondrial and eukaryotic transcription initiation complexes have revealed large-scale conformational transitions in these initiation complexes (24,25).

Here, we use real-time smFRET and alternating laser excitation (26,27) on immobilized RNAP–promoter DNA complexes to reveal substantial large-scale structural dynamics in bacterial transcription initiation complexes. We showed that the transcription bubble in RP_O_ undergoes large-scale structural transitions between different conformations, and identified two long-lived heparin-resistant complexes with transcription-bubble conformations distinct from the open transcription bubble expected for RP_O_. We also show that region σ3.2 stabilizes the open complex conformation. Our results have implications for the mechanisms of promoter opening, and provide a starting point for correlative measurements that monitor the coordination of conformational transitions during promoter recognition and bubble formation.

## MATERIALS AND METHODS

### DNA and protein preparation

Oligonucleotides labelled with Cy3B and ATTO647N were purchased from IBA (Germany) and purified using gel electrophoresis. The purified single-stranded DNA (ssDNA) were annealed in annealing buffer (50 mM Tris–HCl pH 8.0, 1 mM ethylenediaminetetraacetic acid, 500 mM NaCl). All DNA sequences and labelling schemes are shown in Supplementary Figure S1. *Escherichia coli* wild-type (wt) σ^70^, wt RNAP core and ΔJaw RNAP core were prepared as described previously (6,28). *Escherichia coli* σ^70^ with residues 513–519 deleted (Δ3.2) was purified as described previously (29), whereas wt, ΔJaw and Δ3.2 holoenzyme samples were prepared by incubating 50 nM RNAP core with 250 nM σ^70^ for 30 min at 33°C.

### Open complex formation

RNAP-promoter DNA open complexes (RP_O_) were formed as described previously (30–32). Briefly, 10 nM dsDNA was incubated with 50 nM *E. coli* RNAP holoenzyme in T8 buffer (50 mM Tris–HCl pH 8.0, 100 mM KCl, 10 mM MgCl_2_, 100 μg·ml^−1^ bovine serum albumin (BSA), 1 mM dithiothreitol, 5% glycerol) giving a final volume of 20 μl. The mixture was incubated at 37°C for 15 min, after which 1 mg/ml heparin sepharose (GE Healthcare) was added to disrupt non-specific RNAP–DNA complexes. The mixture was incubated at 37°C for 30 s and then centrifuged to remove sepharose beads. The supernatant was then removed and transferred to a pre-warmed Eppendorf tube and incubated for a further 20 min at 37°C.

### Instrumentation

Single-molecule total internal reflection fluorescence (TIRF) experiments were performed on a custom-built objective type TIRF microscope. A green (532-nm Cobolt Samba) and a red (635-nm Cube Coherent) laser were combined using a dichroic mirror and coupled into a fibre optic cable. The fibre output was focused into the back focal plane of the objective (100× oil immersion, numerical aperture 1.4, Olympus) and displaced perpendicular to the optical axis such that laser light was incident at the slide–solution interface at an angle greater than the critical angle, thus creating an evanescent excitation field. Alternating laser excitation (ALEX) was implemented by directly modulating the lasers, and data was acquired using either a 100-Hz or 10-Hz alternation rate (indicated where appropriate). Excitation powers of 3 mW (green) and 1.5 mW (red) were used for 100-Hz experiments, while excitation powers of 1 mW (green) and 0.5 mW (red) were used for 10-Hz experiments. Fluorescence emission was collected by the objective and separated from the excitation light by a dichroic (545/650 nm; Semrock) and cleanup filters (545 mLP, Chroma; and 633/25 nm notch filter, Semrock). The emission signal was focused on a rectangular slit to crop the image and then spectrally separated using a dichroic (630-nm DRLP; Omega) into two emission channels. The channels were focused side-by-side onto an EMCCD camera (Andor iXon 897). The EMCDD was set to an EM gain of 300, corresponding to an approximate real gain of 4.55 counts per photon.

### Sample preparation

Neutravidin-coated glass coverslip chambers were prepared as described (33). A 10 nM solution of biotinylated penta-His antibody (Qiagen) was then incubated for 10 min on the neutravidin-coated surface and excess unbound antibodies were washed away as previously described (34,35). A 1 nM solution of RP_O_ was then incubated on the surface for 5 min followed by washing to remove excess unbound complexes. Imaging buffer containing 50 mM Tris–HCl, 100 mM KCl, 10 mM MgCl_2_, 100 μg·ml^−1^ BSA, 1 mM DTT, 5% glycerol and 2 mM UV-treated Trolox was added to the observation chamber, which was then sealed using a glass coverslip as a lid. An enzymatic oxygen scavenging system consisting of 1 mg·ml^−1^ glucose oxidase, 40 μg·ml^−1^ catalase and 1.0% (wt/vol) glucose was added prior to image acquisition just before sealing the sample. Experiments were performed at 22°C unless indicated otherwise.

### Data analysis

Fluorescence intensities were extracted from images using previously described TwoTone software (36). The apparent FRET efficiency, E*, was calculated as described in (37). We manually inspected intensity time trajectories and selected 100 molecules for analysis according to the following criteria: single-step bleaching after at least 105 frames; circular PSF across DD, DA, and AA channels; no donor photoblinking (although fluctuations were permitted); no acceptor photoblinking (although we allowed for different ATTO647N states; (38)); fluorescence intensities within a limited range; no defocusing; and no nearby molecules as measured using a nearest-neighbour criterion. Histograms of E* were constructed using 250 frames of data from each of our 100 selected molecules; these molecules were collected from independent experiments using a single batch of labeled DNA and a single preparation of RNAP.

We manually classified the intensity time trajectories of individual molecules into groups. Static closed bubble signals were defined as time trajectories showing a stable E*∼0.2 signal with no fluctuations to higher FRET states during our observation period. Static open bubble signals exhibited a stable E*∼0.45 signal with no fluctuations to lower FRET states. Dynamic bubble signals were defined as time-trajectories showing any anti-correlated E* changes during our observation periods.

### HMM analysis of dynamic smFRET time traces and rate determination

Hidden Markov Modelling (HMM) analysis was performed on time-trajectories displaying dynamic FRET fluctuations using custom-written MATLAB software (39,40). Each time-trajectory was fitted with one to three states and the best model (number of states) was selected automatically using maximum evidence criteria (40,41). The states extracted by HMM were categorized according to their FRET efficiency and the states that preceded them; low FRET efficiency states were classified as ‘closed bubble states’, and high FRET efficiency states were classified as ‘open bubble states’. The dwell time distributions for each state category were fitted with an exponential decay curve, which was used to extract the mean open dwell times (τ_open_) and the mean closed dwell times (τ_closed_).

## RESULTS

### Detecting both stable and dynamic intermediates in RNAP–promoter DNA complexes

In previous work, we used solution-based smFRET to study the dynamics of RNAP–promoter DNA complexes (23). Using a FRET pair that reported on transcription bubble formation, we had obtained a FRET histogram with a bimodal FRET distribution. The lower FRET population (E*∼0.2) had been assigned to free promoter DNA and the higher FRET population (E*∼0.4) had been assigned to RP_O_ (23). The width of the RP_O_ population had also been larger than expected merely on the basis of the photon-counting noise of the measurement, with the excess width attributed to conformational heterogeneity. Unfortunately, the short observation window (∼1 ms, characteristic of diffusion studies) of our previous work hindered further study of the heterogeneity, especially at longer timescales (from seconds to minutes).

**Figure 1.**
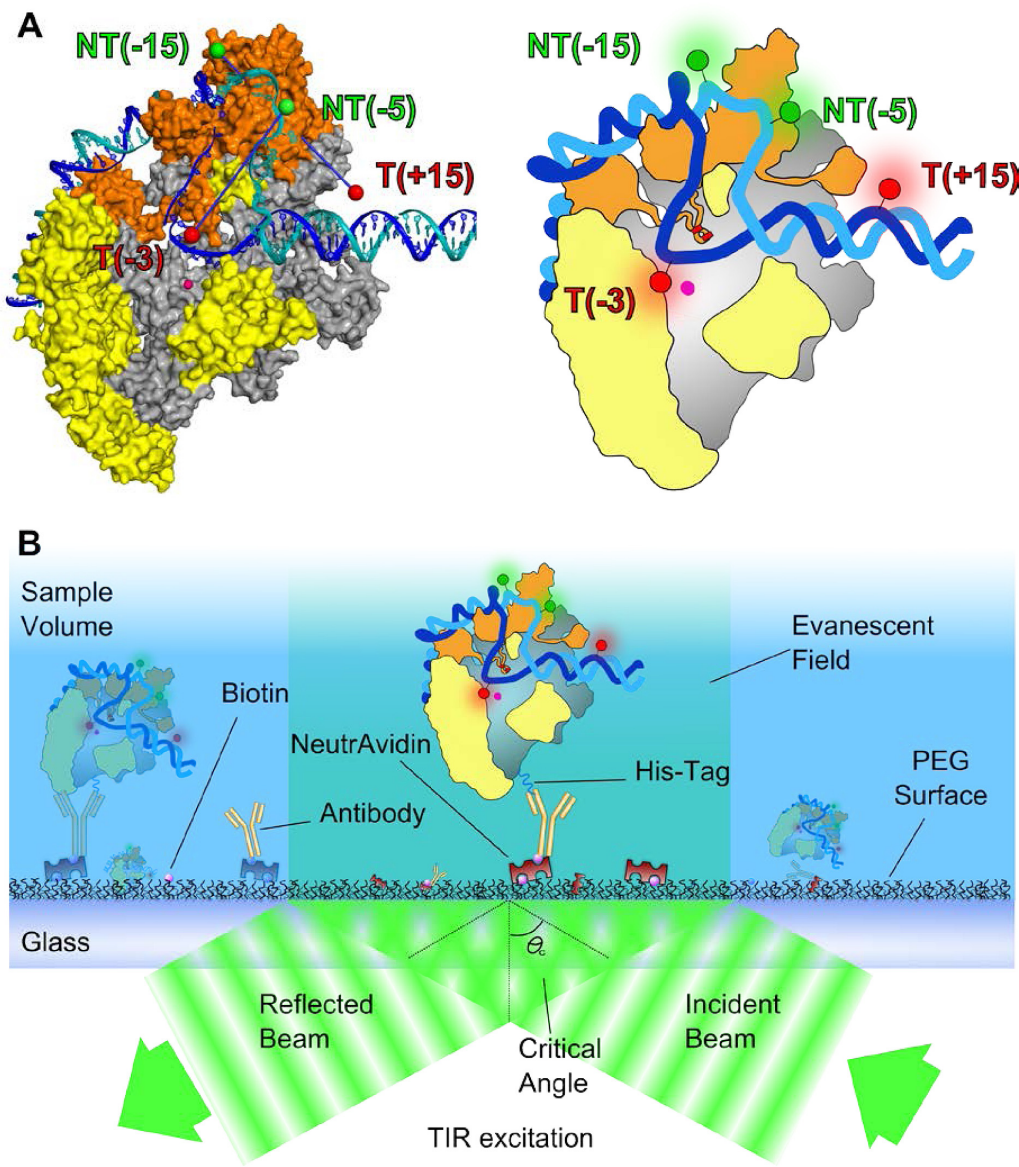
Schematic of experimental strategy used to monitor the transcription bubble conformation. (**A**) Fluorophore positions on a cut-away structural model of *Escherichia coli* RP_O_ obtained using accessible volume modelling (see ‘Materials and Methods’ section). σ^70^ is shown in orange and core protein is shown in grey except for the regions that protrude in front of the cut-away plane, which are yellow. The β subunit is omitted for clarity. The template strand is shown in blue, the non-template strand in teal. The active site Mg^2+^ ion is shown as a pink sphere. (**B**) Schematic of experimental immobilization approach. RP_O_ is immobilized on a PEG-coated surface via anti-His_5_/His_6_-tag interactions.

Here, we examined the presence of RP_O_ dynamics in the 10-ms to 10-sec timescale by using fluorescent substrate DNA and TIRF microscopy to observe immobilized RNAP–promoter DNA complexes for extended periods. Our measurements were performed using a full σ^70^-consensus promoter (lacCONS) based on α *lac* promoter derivative extending from positions −39 to +25 relative to the transcription start site. As in (23), we monitored FRET between a donor and an acceptor fluorophore placed at positions flanking the transcription bubble (23), with the donor incorporated in the −10/−35 spacer DNA (at position −15 of the non-template DNA) and the acceptor on the DNA downstream of the transcription bubble (at position +15 of the template DNA; Figure 1A and Supplementary Figure S1A). These labelling positions have been used extensively in the past and were shown not to interfere significantly with transcriptional activity during initial transcription (23,30,42). The complexes were immobilized via the C-terminus of the RNAP β' subunit (using a C-terminal hexahistidine fusion; Figure 1B) to ensure that any observed stable binding of DNA to the surface was via its contacts with RNAP; this strategy maintains the full activity of RNAP within the transcription complexes (42). Under our conditions, non-specific absorption of free promoter DNA to the surface was negligible (on average, ∼2 molecules per 100 immobilized complexes).

**Figure 2.**
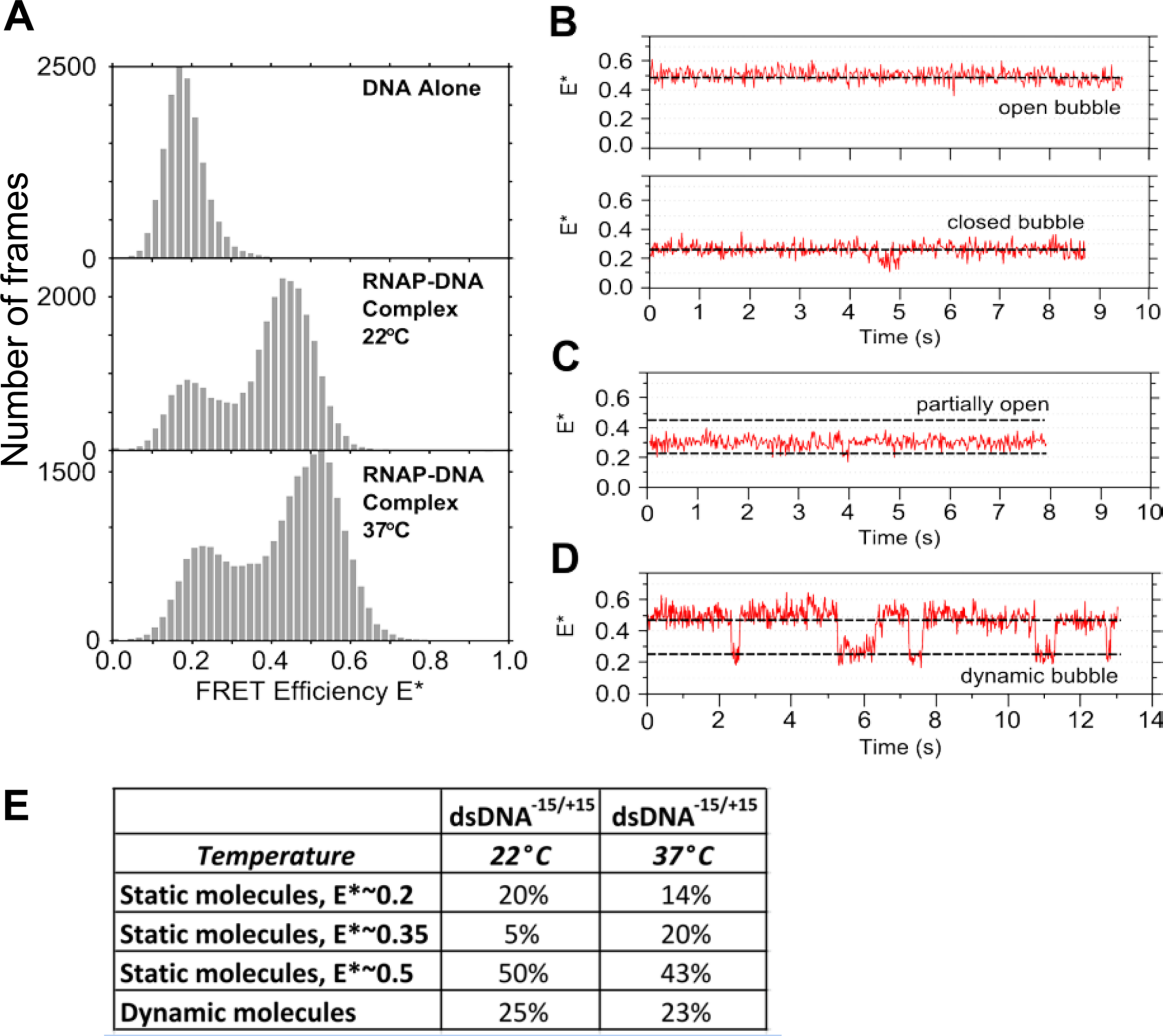
Transcription complexes of RNAP with promoter DNA exhibit substantial structural heterogeneity and dynamics. (**A**) Stacked FRET histograms of promoter DNA (top panel), RNAP–promoter DNA complexes at 22°C (middle panel) and RNAP–promoter DNA complexes at 37°C (bottom panel). (**B**) RNAP–promoter DNA complexes exhibiting either a stable high FRET state (top) or a stable low FRET state (bottom). We assign these as open and closed transcription bubbles, respectively. The FRET signal ends abruptly due to bleaching events. Approximate positions of high and low FRET states are marked with dashed lines. (**C**) RNAP–DNA complex with a stable intermediate FRET value between the expected FRET values for open and closed transcription bubbles. (**D**) RNAP–DNA complex exhibiting dynamic transitions between open and closed bubble conformations. (**E**) Relative abundance of the species formed on dsDNA at 22 and 37°C.

We first performed smFRET measurements on surface-immobilized RP_O_ complexes at room temperature (∼22°C). Our measurements resulted in a FRET histogram showing a major population (∼75%) with a FRET efficiency centred at E*∼0.45, and a smaller population (∼25%) centred at E*∼0.2 (Figure 2A, middle panel). Based on the excellent agreement of the high-FRET population with the FRET efficiency expected for the donor–acceptor pair in RP_O_ (expected E* of ∼0.45, based on the 7.3 nm donor–acceptor distance; Figure 1A), we attributed the high-FRET population to RP_O_ (see also (23)). The low-FRET population had a FRET value similar to that of free dsDNA (E*∼0.17; Figure 2A, top panel); however, since the presence of free DNA has been excluded on the basis of our non-specific adsorption controls, we infer that the conformational state of the promoter DNA in the low-FRET complex more closely resembles the conformation of the promoter DNA within RP_C_, where no substantial melting of the promoter region has occurred.

To study the low-FRET population further, we checked the sensitivity of all complexes to heparin, a non-specific DNA competitor that binds free RNAP and leads to effective removal of any closed RNAP–DNA complexes on lacCONS or lacUV5 promoters (43) upon incubation for 30 or more seconds, The heparin sensitivity was checked by performing FRET experiments on immobilized RP_O_ while using 100 μg/ml heparin in the observation chamber. The addition of heparin did not change the FRET histogram significantly (Supplementary Figure S2), suggesting that the E*∼0.2 population corresponds to a stabilized intermediate formed *after* the heparin-sensitive RP_C_ state.

Similarly, increasing the temperature at which the FRET experiments were performed from 22 to 37°C had only a small effect on the E* distribution, increasing both the peaks of the low- and high-FRET populations by E*∼0.05, but leaving the ratio between the two main subpopulations essentially unchanged (Figure 2A, bottom panel). This indicates that, surprisingly, the E*∼0.2 subpopulation is present even at temperatures where the RP_O_ complex is expected to dominate the conformational equilibrium that includes all intermediates on the path to open complex formation.

To study the heterogeneity source and characterize any dynamics, we inspected individual time-traces of immobilized complexes. Inspection of traces from several molecules (*N* = 100) revealed the presence of additional heterogeneity, with complexes exhibiting four characteristic behaviors. Three behaviors involved single FRET states that appeared ‘static’ (i.e. not exhibiting any clear transitions to well-separated, stable FRET states): 50% of the molecules exhibited a stable E*∼0.45 state (Figure 2B, top), ∼20% exhibited a stable E*∼0.2 state (Figure 2B, bottom), and ∼5% displayed a stable E*∼0.35 state (Figure 2C), which was not apparent in the middle and bottom E* histograms of Figure 2A due to its low amplitude.

The fourth population, comprising ∼25% of the traces, displayed dynamic FRET fluctuations within the 10-s acquisition times, either between the 0.2 and 0.45 FRET states (Figure 2D), or between the E*∼0.35 state and the other two states (∼0.45 or ∼0.2; Supplementary Figure S3). This observation strongly suggests that the E*∼0.35 FRET state is a real structural state (as opposed to a state that reflects trivial baseline shifts in our FRET signal), which we tentatively assign to a partially open transcription bubble (Figure 2E).

We also examined time-traces from our experiments at 37°C (Figure 2E and Supplementary Figure S4). Although there is no significant change in the number of molecules exhibiting dynamics (25% at 22°C, 23% at 37°C), there is a large increase in the E*∼0.35 population, from 5% at 22°C, to 20% at 37°C.

### RNAP–promoter DNA complexes exhibit bubble opening-closing dynamics

To provide another vantage point for monitoring dynamics within transcription complexes, and especially within the transcription bubble, we placed FRET fluorophores within the transcription bubble by employing a −5/−3 labeled promoter DNA (Supplementary Figures S1C and 5) previously used to monitor bubble opening (63). In the closed bubble conformation, close donor–acceptor proximity is expected either to lead to contact-mediated quenching (which will suppress fluorescence from the FRET pair) or to FRET species with >95% FRET (since close proximity between fluorophores and a protein can prevent contact-mediated quenching). As the DNA strands separate as the bubble opens, a FRET efficiency of E*∼0.65 is expected (23,63) (Supplementary Figure S5A).

We performed experiments on immobilized complexes at 22°C and identified molecules exhibiting dynamic behaviour, in which the FRET signal fluctuates between a predominant FRET state centred at E*∼0.6 and a very high FRET state (E* > 0.8; Supplementary Figure S5B). As this DNA directly reports on the proximity of the template and non-template strands in the transcription bubble, this result supports our suggestion that the FRET fluctuations in Figure 2D correspond to structural transitions in which at least part of the transcription bubble opens and closes.

**Figure 3.**
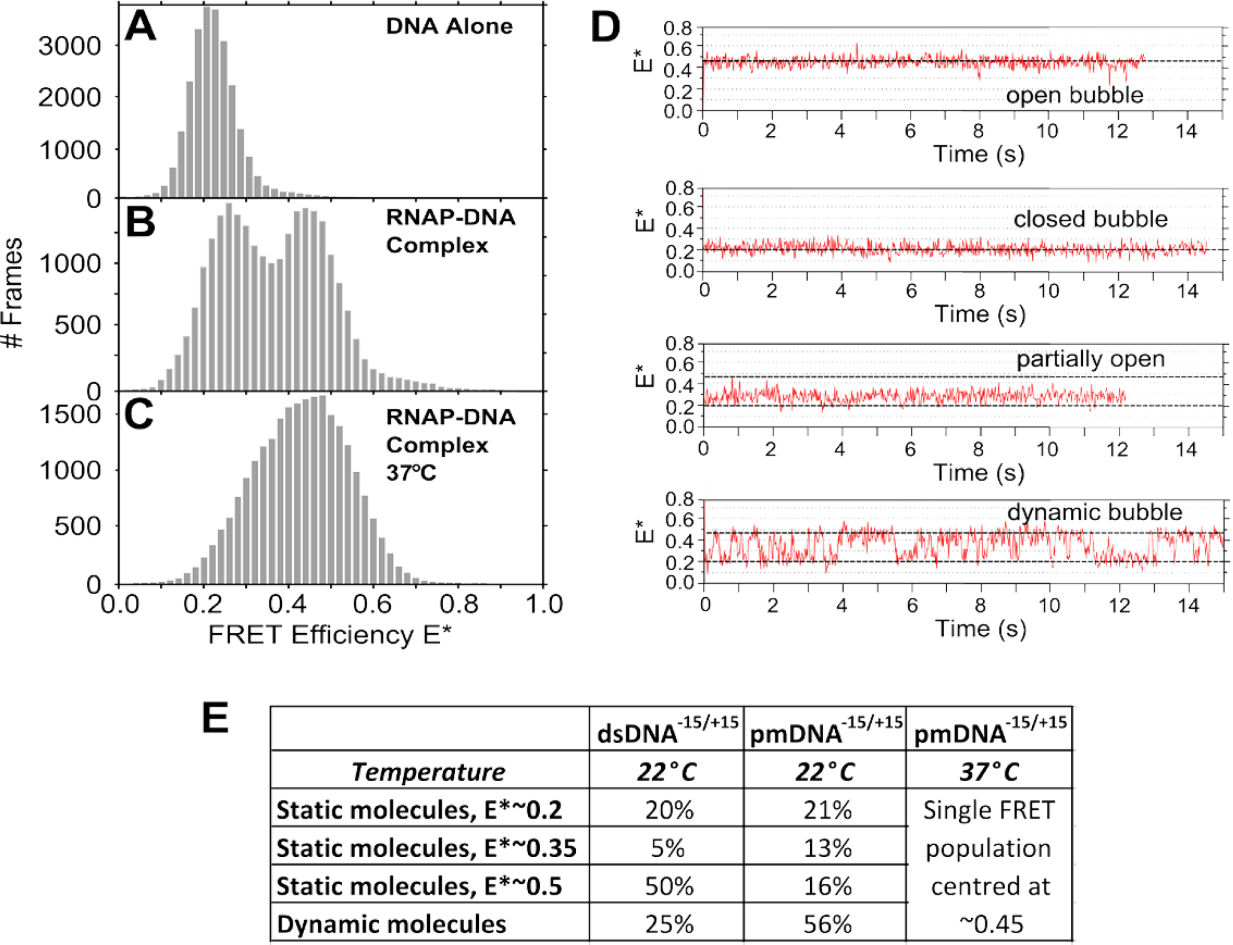
Effect of the transcription bubble mismatch on the transcription bubble conformational landscape. (**A**) Stacked FRET histograms of promoter pmDNA only at 22°C. (**B**) RNAP–promoter pmDNA complexes at 22°C. (**C**) RNAP–promoter pmDNA complexes at 37°C. (**D**) Timetraces of the four main species observed using pmDNA at 22°C. (**E**) Relative abundance of the species formed on pmDNA at 22 and 37°C.

One possible explanation for the observed dynamics is that some RNAP molecules are incapable of stable promoter opening. We tested this hypothesis by performing smFRET experiments using a pre-melted promoter DNA fragment (pmDNA) with dyes positioned at +15 and −15 (Supplementary Figure S1B). By placing mismatches within the transcription bubble (from positions −10 to −4 relative to the transcription start site), we mimic bubble nucleation and bias the transcription initiation reaction toward the RP_O_ state (44,45). For the pmDNA only, we observed a FRET state centred at E*∼0.2 (Figure 3A); however, RNAP addition led to a bimodal distribution (E*∼0.26 and E*∼0.45) similar to that observed for the fully double-stranded DNA (Figure 3B). The small shift of the low-FRET distribution (by 0.06 FRET units) may be attributed to differences in the degree of bubble opening in the low-FRET distributions for the two different DNAs since the −10/−4 part of the bubble is premelted for pmDNA.

Comparison of time-traces of pmDNA at 22°C (Figure 3D and E) with thosefor dsDNA shows a significant increase in the intermediate complex for pmDNA (from 5 to 13%). Further, there is a large increase in the number of molecules showing dynamics (from 25 to 56%). However, static FRET complexes are still present for pmDNA (with 21% showing low FRET, and 16% showing high FRET).

Increasing the temperature from 22 to 37°C for the pmDNA produced a single population centred at E*∼0.45, which showed an increased width compared to the 0.45 population at 22°C (Figure 3C); the increased width reflects fast, unresolved dynamics between the 0.45 population and a lower-FRET state (Supplementary Figure S4E and F). There was also a complete absence of an E*∼0.20 FRET state, suggesting that the −10/−4 mismatch and the increased temperature shifts the equilibrium to the open state, and indicates that the E*∼0.2 state may correspond to an intermediate that occurs ahead of initial transcription bubble melting.

### Region σ3.2 and β’ jaw influence the conformations and dynamics of RNAP–promoter complexes

**Figure 4.**
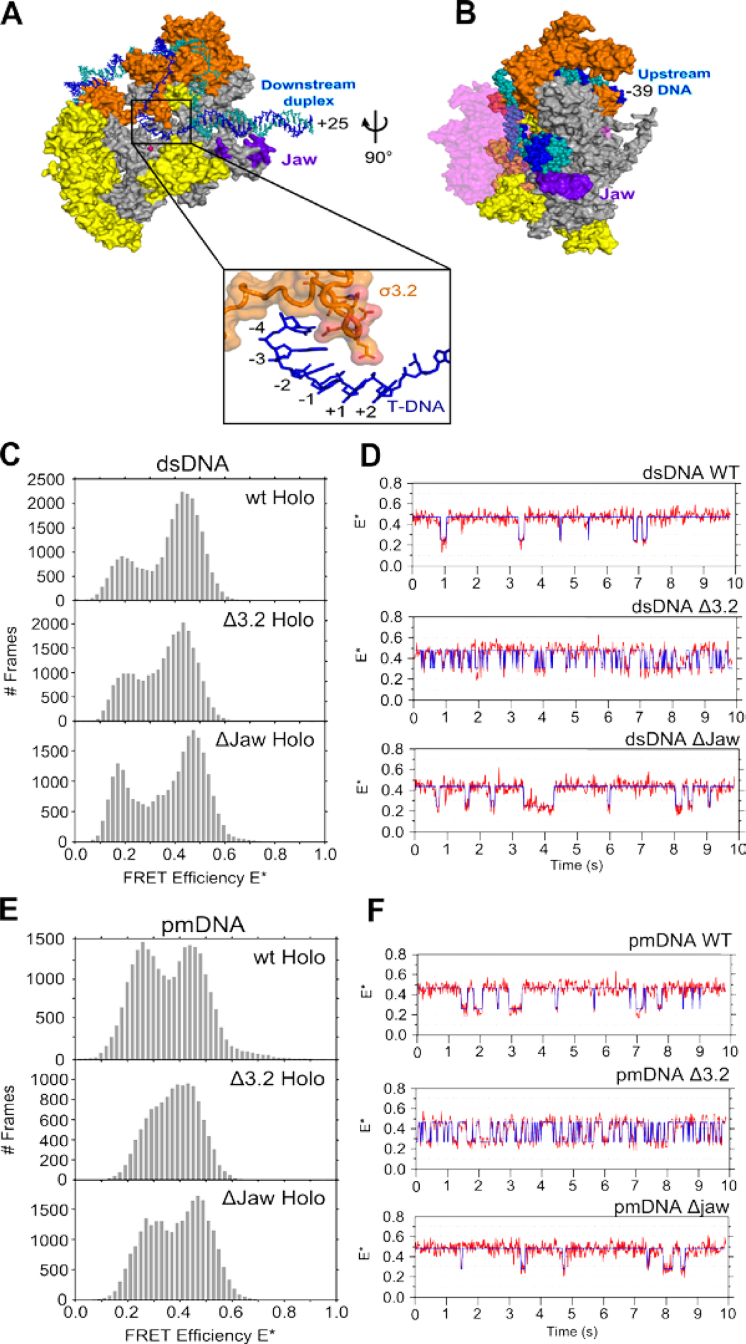
Structures of the σR3.2 finger and β’ jaw. (**A**) Top figure shows a cut-away structural model of *Escherichia coli* RP_O_. σ^70^ is shown in orange and core protein is shown in grey except for the regions that protrude in front of the cut-away plane, which are yellow. The β subunit is omitted for clarity. The template strand is shown in blue, the non-template strand in teal. The jaw subdomain region that is deleted in this study is labelled and shown in purple. The region in the box is a zoomed view of the RNAP active site that is marked in the full RP_O_ model and shows the interactions made between the σR3.2 finger and the transcription bubble template strand. The side chains of σR3.2 residues deleted in this study are shown. (**B**) Zoomed view of the open complex after it has been rotated 90° in the direction of the curly arrow. This view shows the close contacts made between the jaw and downstream DNA. The β subunit is shown partially transparent and coloured pink. (**C**) Stacked FRET histograms of wt (top), Δ3.2 (middle) and ΔJaw (bottom) complexes made using dsRNA. (**D**) Example time trajectories of wt, Δ3.2 and ΔJaw RNAP–dsDNA complexes displaying bubble opening and closing transitions. The data have been fit to a Hidden Markov Model (blue line). (**E**) Stacked FRET histograms of wt (top), Δ3.2 (middle) and ΔJaw (bottom) complexes made using pmRNA. (**F**) Example time trajectories of wt, Δ3.2 and ΔJaw RNAP–pmDNA complexes displaying bubble opening and closing transitions. The data have been fit to a Hidden Markov Model (blue line).

We next examined whether two RNAP structural regions, the σ^70^ region 3.2 (σ3.2) and the β’ jaw, influence the profile of transcription complexes. Both regions play important roles in transcription initiation. For example, crystal structures of *Thermus thermophilus* RNAP–promoter complexes showed that σ3.2 forms a loop that protrudes towards the RNAP active centre, where it interacts with the template strand (Figure 4A) (18,46); such interactions may help σ3.2 to correctly position the template within the RNAP active site cleft (15,47), and to stabilize the transcription bubble (29). The β’ jaw is thought to position the non-template strand in the DNA binding cleft (Figure 4B) and stabilize RP_O_ by assembling on the DNA downstream of the transcription start site late in the RP_O_ formation pathway; consistent with this, jaw deletions reduced the RP_O_ lifetime at various promoters (48), and stabilized an RP_O_ intermediate at the λP_R_ promoter (6). We thus hypothesized that the absence of either σ3.2 or the β’ jaw may destabilize the transcription bubble open conformation.

To test our hypothesis, we first studied an RNAP holoenzyme containing a σ^70^ variant that lacks amino acids 513–519 (Δ3.2), a region that corresponds to the tip of the σ3.2 loop. We analyzed RP_O_ complexes made using fully complementary DNA and recovered a bimodal E* distribution with peaks similar to that of wt RNAP–promoter dsDNA complexes (centred at E*∼0.2 and ∼0.45) (Figure 4C, top panel); however showing slightly increased intermediate E* values (E*∼0.35) (Figure 4C, middle panel).

We also studied an RNAP containing a β’ subunit variant that lacks the jaw subdomain (amino acids 1149–1190; ΔJaw), which showed a similar FRET distribution compared to that for wt RNAP (Figure 4C, bottom panel). The greater E*∼0.35 intermediate FRET population may reflect either faster interconversions between the E*∼0.2 and ∼0.45 subpopulations, or an increase in the E*∼0.35 subpopulation. Analysis of time trajectory data (Figure 4D) revealed that the increased intermediate E* values for Δ3.2 were largely due to faster interconversions relative to wt complexes (cf. top and middle panels; see also next section), supporting the hypothesis that σ3.2 anchors the template DNA on the rest of RNAP and influences the conformational dynamics of the transcription bubble.

Next, we studied the FRET distributions of Δ3.2 RNAP complexes formed using pmDNA (Figure 4E). Here, the two main FRET peaks merge into a wider distribution biased towards the open-bubble conformation (E*∼0.45), as compared to the corresponding FRET histogram for wt RNAP (Figure 4E, compare top and middle panels). Similar experiments with ΔJaw complexes showed little difference compared to wt (Figure 4E, bottom panel). Analysis of individual time traces again showed that Δ3.2 complexes exhibited much faster dynamics than wt complexes (Figure 4F; cf. top and middle panels).

### Kinetic analysis of interconversions within dynamic complexes

To measure the rates for the transitions between the low- and high-FRET states for the dynamic complexes, we analyzed individual time-traces for wt, Δ3.2 data and ΔJaw complexes. After obtaining dwell time distributions for the low- and high-FRET states using HMM analysis, we fitted them to exponential functions, and quantified the interconversion rates, which we interpret as bubble opening and closing rates (see ‘Discussion’ section).

**Figure 5.**
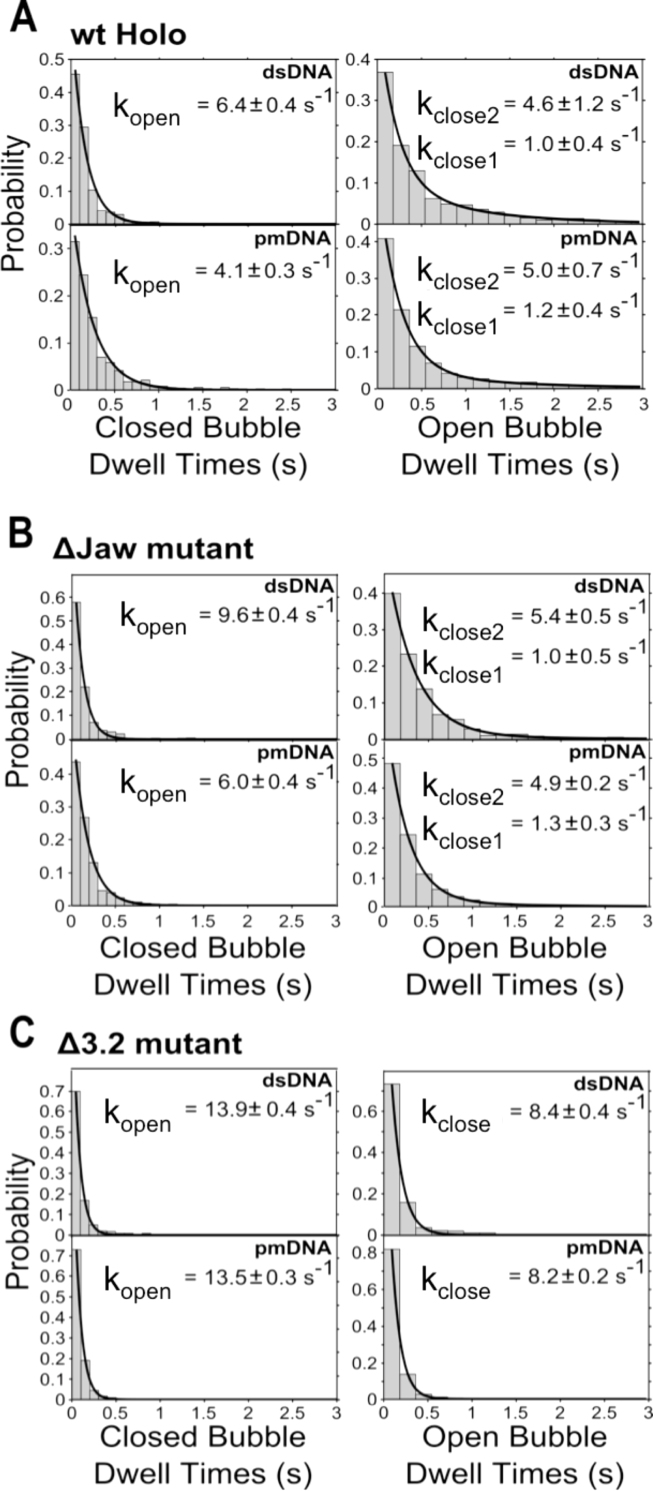
Effect of targeted RNAP deletion mutations on the kinetics of the transcription bubble conformation. (**A**) Stacked histograms of closed (left) and open (right) bubble conformation dwell times for dsDNA and pmDNA with wt RNAP. The data are fitted to either single- or double-exponential functions, and the bubble opening and closing rates are shown inset. (**B**) Stacked histograms of closed (left) and open (right) bubble conformation dwell times for dsDNA and pmDNA with ΔJaw RNAP. (**C**) Stacked histograms of closed (left) and open (right) bubble conformation dwell times for dsDNA and pmDNA with Δ3.2 RNAP.

For RNAP–promoter dsDNA complexes, we found that the low-FRET state (E*∼0.2) exhibited a mean lifetime (dwell time) of ∼150 ms, which corresponds to an opening rate k_open_ of 6.4 s^−1^ (Figure 5A, top left panel). In comparison, the closing transition was more complex, following bi-exponential decay kinetics, with the open bubble state exhibiting mean lifetimes of ∼220 ms and ∼1 s (Figure 5B, top right panel). For all conditions tested, the bubble mismatch (pmDNA) did not influence the high-FRET lifetimes (Figure 5A, bottom right panel), but increased the mean lifetime of the low-FRET state (∼240 ms; Figure 5A, bottom left panel); the latter effect may be due to the mismatch increasing the energetic barrier for the transitions that ultimately lead to bubble opening.

Deletions in the jaw region of the β’ subunit of RNAP using both dsDNA and pmDNA decreased the average lifetimes compared to wt, corresponding to slightly longer opening and closing rates (Figure 5B). This suggests that the Δjaw mutation has a small effect on the stability of the high-FRET species. In comparison, the effect of deleting σ3.2 was much more significant, with bubble opening and closing rates increasing 2- to 4-fold for Δ3.2 complexes compared with wt complexes (Figure 5C). This finding supports the hypothesis that the σ3.2 finger influences the positioning of the template strand at the active centre. In addition, the presence of the initiating dinucleotide (ApA; complementary to the first 2 nt on the template strand), which stabilizes RP_O_, was found to have little effect on the kinetics of interconversion for the dynamic molecules (Supplementary Figure S6).

## DISCUSSION

Previous work on transcription initiation had shown that RP_O_ formation proceeds with a multi-step mechanism involving intermediates whose exact structural details are promoter-dependent. Here, we extend our understanding of initiation by providing insight into RP_O_ formation and the role of RNAP structural elements in promoter melting. Our single-molecule work complements several high-resolution structures of complexes related to open complex formation (8,18,46,49–52), as well as ensemble biochemical work. Specifically, we address challenges due to difficulties in capturing transient intermediates, due to lack of synchronization, and due to the potential presence of many reaction paths (e.g., off-pathway species) towards the open complex. Our results also agree with previous studies showing that varying conditions, such as temperature, can bias the conformational equilibrium towards distinct structural intermediates in the RP_O_ formation pathway (12,53,54).

### Open complexes are conformationally heterogeneous and dynamic

By monitoring individual complexes for many seconds, we identified considerable conformational heterogeneity and dynamics among transcription complexes. Our results clearly establish that, under conditions where one expects to observe stable RP_O_ almost exclusively, we can see additional complexes.

To our knowledge, this is the first study that identifies three distinct heparin-resistant RNAP–promoter DNA complexes existing as a heterogeneous mixture at a *lac* promoter derivative. Intriguingly, this is the case even at 37°C, where RP_O_ should be the dominant species by far. Notably, this heterogeneity has remained hidden both in ensemble studies, as well as in single-molecule confocal studies on diffusing molecules (23); specifically, due to the presence of substantial amount of free DNA in the solution containing RP_O_ complexes (as a result of heparin challenge), the latter studies could not distinguish between free DNA and transcription complexes with a similar FRET signature. Here, all DNA molecules observed on the surface are retained via long-lived interactions with surface-immobilized RNAP molecules, and thus our experiments do not contain any free DNA.

### Assigning static FRET species to complexes along the path of open complex formation

Using the FRET signatures as a guide, and supported by the dependence of the states on temperature, competitors and mutations, we assigned the observed structural states to initiation complexes exhibiting different degrees of transcription bubble opening: a first state in which the bubble is likely to be either closed or in the very early stages of opening (E*∼0.2), a second where the bubble is likely to be fully open (E*∼0.45), and a third with a FRET signature intermediate to those of the open- and closed-bubble states (E*∼0.35).

The exact nature of our static E*∼0.2 complex is unclear. One possibility is that it resembles the RP_i_ intermediate reported in *lac*UV5 (11), an ‘advanced’ closed complex with resistance to poly(dA:dT) (a non-specific competitor for RNAP binding, similar to heparin), a bubble in a closed conformation and increased abundance below 22°C. Another candidate is a heparin-resistant form (possibly due to the very tight promoter interactions of lacCONS with RNAP) of the advanced closed complex I_1, L_, which has been observed on λP_R_ and has been shown to be heparin-sensitive (5). In such a complex, one face of the downstream DNA duplex is protected by RNAP up to position +20, possibly by the DNA being inserted in the RNAP main cleft. Indeed, the static E*∼0.2 complex we observed is heparin-resistant, and has a FRET signature identical to closed free dsDNA; further, the complex is present in significant amounts both at 22 and 37°C. A second possibility for the static E*∼0.2 complex is that it resembles a less advanced (but still quite stable) closed complex, since it is missing DNA upstream of position −40; in λP_R_, the absence of the upstream region shortened the RNAP footprint on DNA from +20 to +7.

Our static E*∼0.45 complex is likely to represent a stable RP_O_ on *lac*UV5, featuring a fully open bubble. This assignment is based on the fact that the RP_O_ should be the predominant species under the conditions of our experiments (strong *lac* consensus promoter, temperature of 22 and 37°C, and enough time to equilibrate and form RP_O_ prior to microscopy).

The nature of the relatively rare E*∼0.35 state is unclear. We speculate that it is an intermediate with a partially open or nucleated transcription bubble, similar to the intermediate O_l_ identified at the *lac*UV5 promoter: the degree of promoter unwinding in O_l_ was different from that of the O_h_ complex, which was the more abundant species at 37°C and presumably has a fully open transcription bubble. Alternatively, the E*∼0.35 state could represent scrunched or anti-scrunched promoter conformations that shift the transcription start site in OC more downstream or upstream, respectively (55,56). Further work, including experiments that monitor structural states populated during real-time formation of the open complex should shed more light on the nature of this minor state.

Our results are likely to be affected by the absence of DNA upstream of position −40, which has significant effects on the kinetics and equilibria involved in RP_o_ formation on promoters *lac*UV5 and λP_R_ (5,57,58), with the main effects being the stabilization of the first kinetically significant intermediate (I_1_ for λP_R_ and RP_i_ for lacUV5) and the large decrease of the isomerization rate k_2_ for RP_o_ formation. Specifically for *lac*UV5 (57), k_2_ decreased 10- to 50-fold (relative to full-length fragments) for short fragments ending at −42 or −45, and the equilibrium constant K_1_ for the formation of RP_c_ increased by 10-fold (for native RNAP). Both effects are expected to stabilize a heparin-resistant closed complex that we assign to our static 0.2 FRET species at 22°C. The work by Ross *et al.* (57) also clearly shows that the stability and KMnO_4_ footprints of RP_O_ complexes on short fragments match those formed on DNAs extending up to −130, strongly suggesting that RP_o_ complexes on short promoter fragments have a very similar structure to those on longer promoter fragments; the difference lies mainly in the relative abundance of intermediates due to changes in rates.

### Direct observation of reversible promoter opening and closing

We also observed that many RNAP–promoter DNA complexes undergo large FRET changes, similar to those observed for mitochondrial and eukaryotic transcription initiation complexes (24,25). Surprisingly, our results showed that bubble transitions persist and, moreover, increase when we form complexes using pre-melted DNA, suggesting that the dynamic behavior is not due to deficient transcription bubble opening, but rather, an intrinsic feature of RNAP–promoter DNA complexes.

Significantly, only 25% of the complexes show FRET fluctuations, a fact that points to high energy barriers separating these fluctuating complexes from static states (i.e. the E*∼0.2 and 0.45 complexes) and to a complex conformational landscape which contains slow conformational transitions (slower than 10 s) inaccessible to our current FRET rulers. The absence of larger differences in the transition kinetics between the ΔJaw mutant and wtRNAP suggest that the observed FRET transitions are not due to the repositioning of the ‘downstream mobile element’ (a number of RNAP regions including the β’ jaw; (21)).

What are the states formed during the opening/closing transition? The similarity in the kinetics of the dynamic molecules between dsDNA and pmDNA shows that the main rate-limiting steps in both cases is the opening and re-closing of the *downstream* end of the transcription bubble (i.e. melting of the −3/+2 region). This means that the low-FRET dynamic state in both dsDNA and pmDNA is partially open, whereas the high-FRET dynamic state is the first complex with a fully open bubble. The latter is often referred to as intermediate I_2_ (using the naming convention in Ruff *et al.* (21)) and requires large conformational changes in RNAP to reach the stable open complex RP_O_; these changes involve movements of the RNAP downstream mobile element. At 37°C, the additional thermal energy favours the opening process, leading most complexes to show the E*∼0.45 signature.

### Implications for the mechanism of promoter opening

The direct observation of transcription bubble dynamics has implications for addressing the long-standing question of the sequence by which promoter unwinding and loading into the DNA-binding cleft occurs. Two main models exist: *bend-load-open* and *open-bend-load* (59). In the *bend-load-open* model, full DNA unwinding occurs after the duplex DNA has entered the DNA-binding cleft and is promoted by interactions between the clamp and downstream DNA (53). In the *open-bend-load*, DNA unwinding must occur before entering the binding cleft (13).

Our work shows that the transcription bubble readily undergoes conformational fluctuations once a stable complex has formed. Furthermore, experiments investigating the RP_O_ clamp conformation showed that the clamp appears to remain 100% closed even when the bubble is expected to undergo opening and closing transitions (19). This indicates that at least the downstream bubble opening and closing (from positions −3 to +2) can occur when promoter DNA is located *inside* the DNA binding cleft, consistent with the *bend-load-open* model. Our observations also support the notion that full DNA melting does not need to occur prior to loading into the binding cleft (5). Another possible interpretation is that the downstream part of the non-template bubble DNA can exit from the main RNAP cleft even when the RNAP clamp is closed.

An alternative explanation of our results along the lines of the *open-bend-load* model and in the context of the conformational equilibrium of the clamp (19) will require full DNA opening prior to loading, and a transient opening and rapid reclosing of the clamp to allow loading of the melted DNA. However, separate work in our laboratory on the real-time clamp dynamics in RP_O_ shows no detectable transitions within 20 ms (our temporal resolution), rendering the *open-bend-load model* less likely (Duchi *et al.*, in preparation).

The fact that the Δ3.2 RNAP–DNA complexes exhibit faster bubble dynamics supports proposals based on structural studies that σ3.2 stabilizes the template strand at the active site, and raises the intriguing possibility that the altered opening and closing kinetics might influence later steps in initiation (see next section). Our results do not show a significant role for the β’ jaw in controlling bubble dynamics; however, they do suggest that the jaw influences the degree of transcription bubble opening, in agreement with earlier studies (6).

### Possible functions of static and dynamic heterogeneity

The fact that the initiating dinucleotide ApA—which is expected to stabilize the template strand in one particular register at the active site (23)—does not influence bubble opening and closing kinetics, suggests that the bubble structural transitions do not reflect attempts by RNAP to place the template strand at the ‘correct’ start site. However, the presence of various RNAP–romoter DNA complexes may influence gene expression by affecting later stages of transcription initiation. For example, different bubble conformations could influence promoter escape in a manner similar to that reported in (54), where an O_l_ intermediate was found to produce more abortive products and be less efficient at promoter escape than an O_u_ complex. Further, in (60), RNAP–promoter DNA complexes were found to be more likely to escape the promoter if interactions between the polymerase and promoter DNA were weak. Similar observations apply to ‘moribund complexes’, a subpopulation of transcription complexes that produce abortive products but are unable to escape from the promoter (61). Since states other than the fully open complex are present at 37°C, we speculate that they may reflect relevant physiological conformations related to altered transcription initiation and promoter-escape properties. Further support for this hypothesis comes from recent biochemical work on several promoters (λP_R_, T7A1 and *rrnB*) that showed the presence of open complex subpopulations associated with different properties in terms of initiation transcription and promoter escape (62); specifically, it was shown that long-lived open complexes (e.g., on λP_R_) produced longer abortive products than short-lived open complexes (e.g. on T7A1), which instead produced shorter abortive products.

In future work, we will use our single-molecule FRET assay to monitor transcription initiation from the initial DNA binding event to promoter escape. Such studies will allow us to determine whether the conformational states we observe are sequential intermediates along the RP_O_ formation pathway, to determine whether the initial heterogeneity persists in later stages (such as in abortive initiation and promoter escape), and to identify additional DNA and RNAP determinants of transcription complex heterogeneity and dynamics.

## Supplementary Material

Supplementary Data
