# Supplementary material for: Conformational heterogeneity and bubble dynamics in single bacterial transcription initiation complexes
Source: Nucleic Acids Res. 2017 Nov 21;46(2):677–88. doi: 10.1093/nar/gkx1146 (PMC5778504; doi:10.1093/nar/gkx1146)
Supplement: Supplementary Data [file gkx1146_supp.pdf]

Supplementary Information for

Conformational heterogeneity and bubble dynamics in single bacterial transcription initiation complexes

Diego Duchi[1], Kristofer Gryte[1], Nicole C. Robb[1], Zakia Morichaud[2], Carol Sheppard[3], Konstantin Brodolin[2], Sivaramesh Wigneshweraraj[3] and Achillefs N. Kapanidis[1,*]

[1]Gene Machines Group, Biological Physics Research Unit, Clarendon Laboratory, Department of Physics, University of Oxford, Oxford, OX1 3PU, United Kingdom

[2]CNRS UMR 9004, Institut de Recherche en Infectiologie de Montpellier, Université de Montpellier, 1919 route de Mende, 34293 Montpellier, France

[3]MRC Centre for Molecular Microbiology and Infection, Imperial College London, London SW7 2AZ, UK

[*]To whom correspondence should be addressed. Tel: +44(0)1865272226; Email: a.kapanidis1@physics.ox.ac.uk

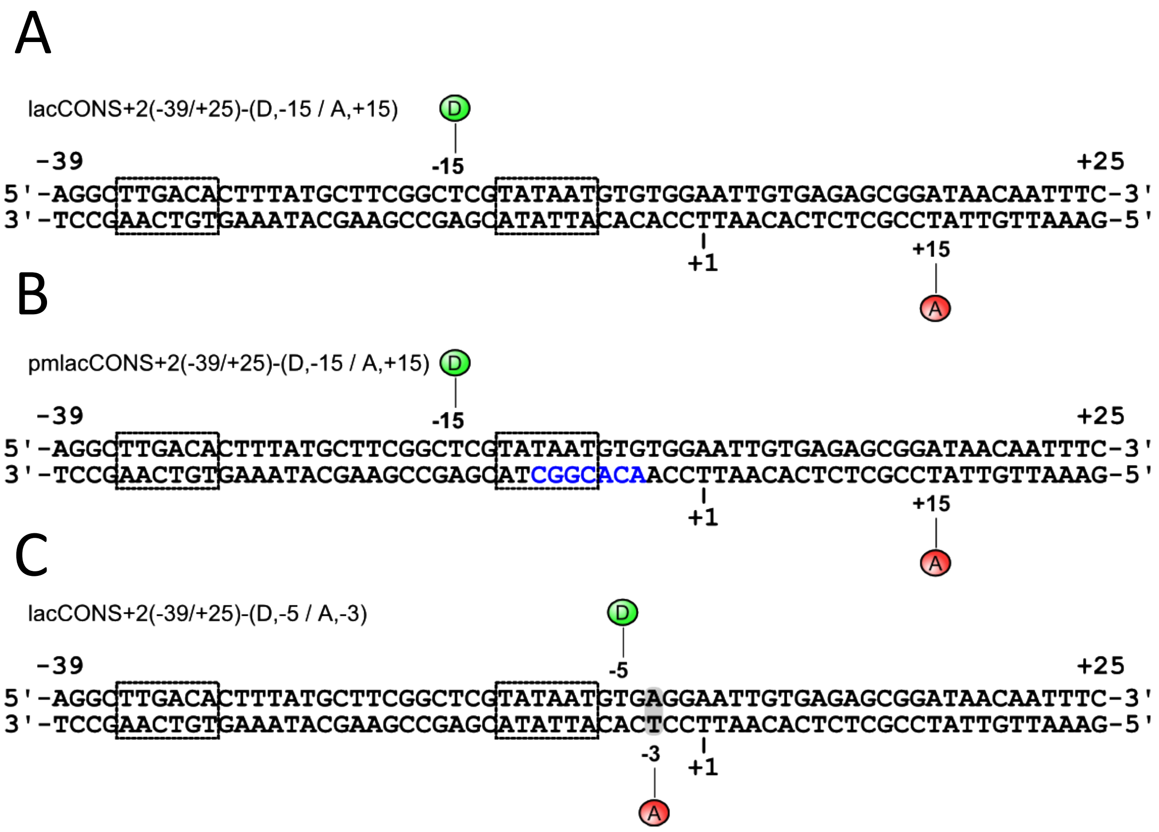

Figure S1. Sequences and labelling positions of DNA. The -35 and -10 promoter elements are shown in boxes. The transcription start site (+1) is marked, as are the fluorophore labelling sites. (A) Fully complementary promoter DNA. (B) Pre-melted promoter DNA with mismatch shown in blue. (C) ‘Bubble ruler’ DNA. The -3 position has an inversion mutation (shaded grey) to permit the labelling of the -3 position. In all DNA fragments, the donor is Cy3B, the acceptor is ATTO647N.

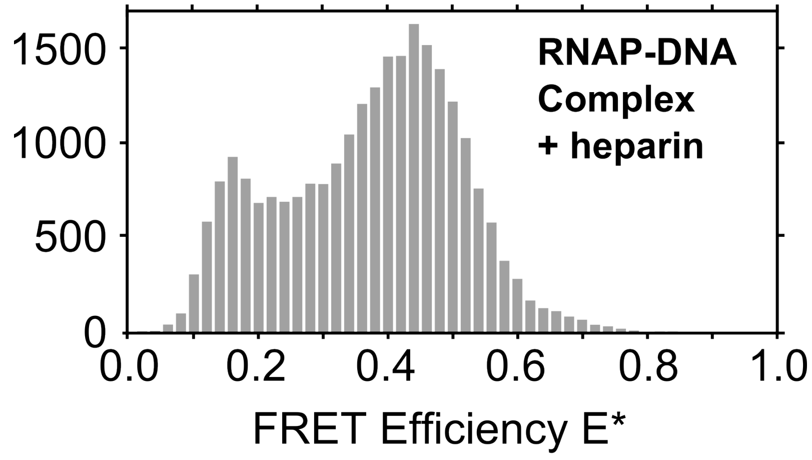

Figure S2. FRET heterogeneity does not change upon heparin addition. FRET histograms of RNAP-promoter DNA complexes at 22°C in the presence of 100 µg/ml heparin in the observation chamber.

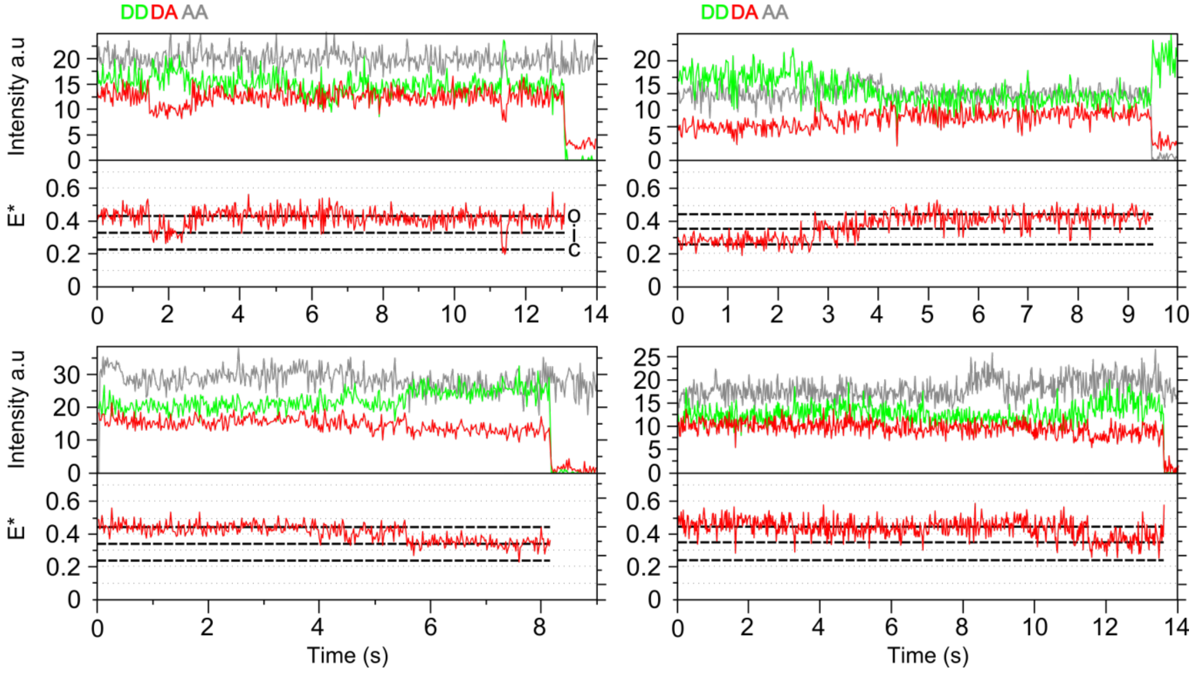

Figure S3. FRET transitions involving the E*~0.35 state. Example time trajectories of RNAP-promoter DNA complexes displaying transitions between E*~0.35 and E*~0.45, and between E*~0.35 and E*~0.20 states. Fluorescence intensity time trajectories show that the FRET changes are anti-correlated and not due to fluorophore photophysical aberrations. The dashed lines indicate the approximate FRET values of the three major FRET states observed in our experiments.

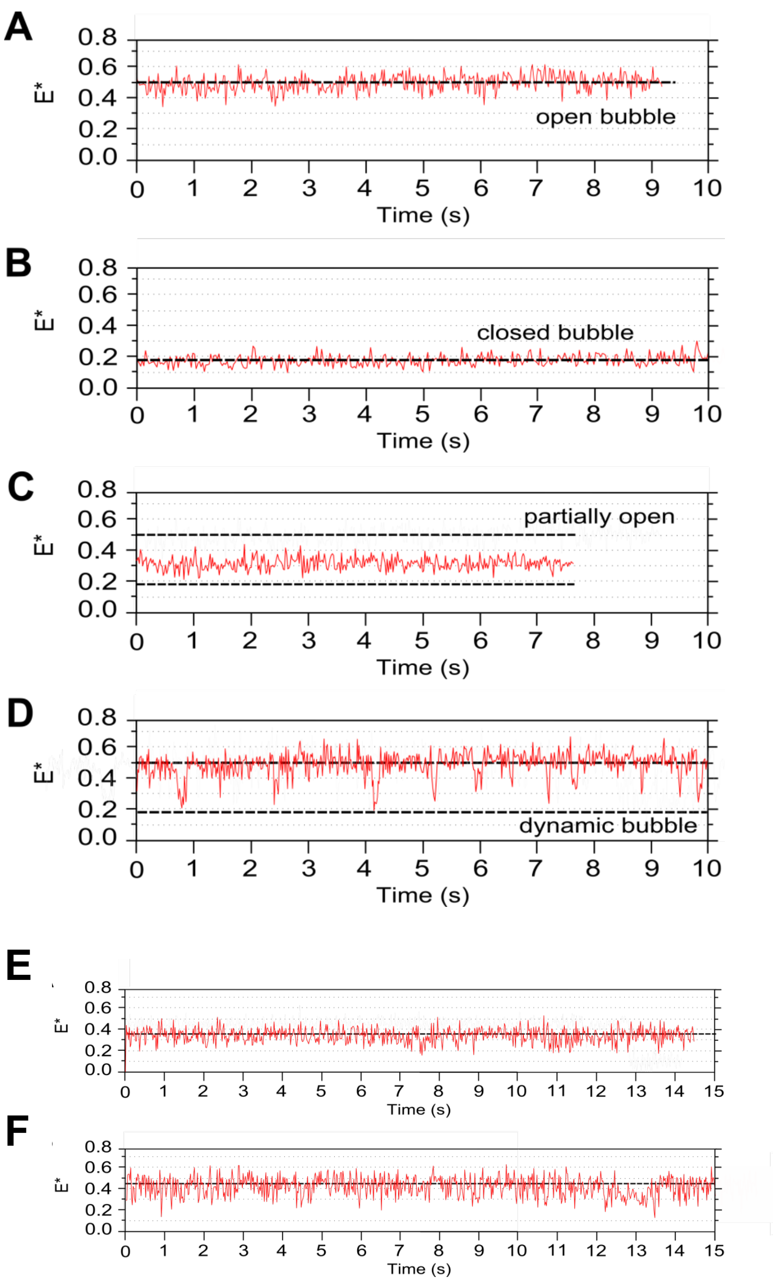

FIGURE S4. FRET time-traces for RNAP-DNA complexes incubated at 37°C. A-D. Examples of time trajectories of static and dynamic RNAP-promoter DNA complexes formed on double-stranded promoter DNA. **E-F**. Example of time trajectories of RNAP-promoter DNA complexes formed on pre-melted promoter DNA.

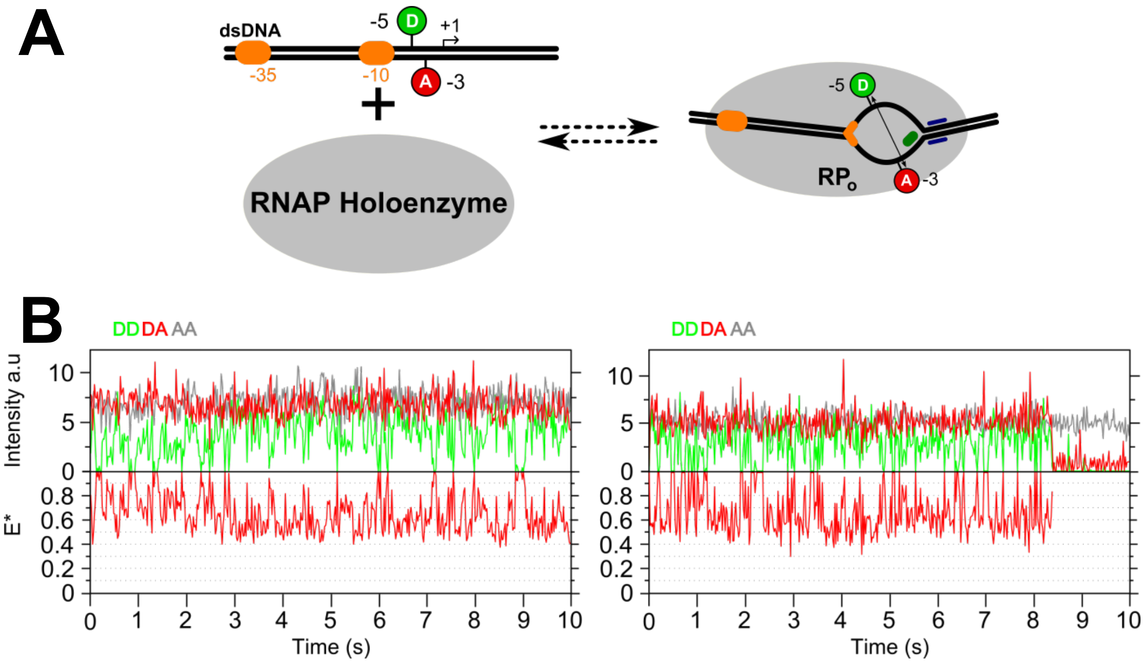

Figure S5. FRET transitions using the bubble ruler promoter DNA pointing to open-close bubble dynamics. (A) Schematic showing the formation of RP_O using the bubble ruler DNA. The donor and acceptor fluorophores are in close proximity in dsDNA and move further apart in RP_O. The positions of the active site (green oval), the binding cleft (blue), the -10 element, and the -35 element are shown. (B) Example time trajectories of complexes formed using the bubble ruler. Clear transitions occur between a high-FRET state (E*>0.8) and an intermediate FRET state consistent with the FRET pair separation in RP_o.

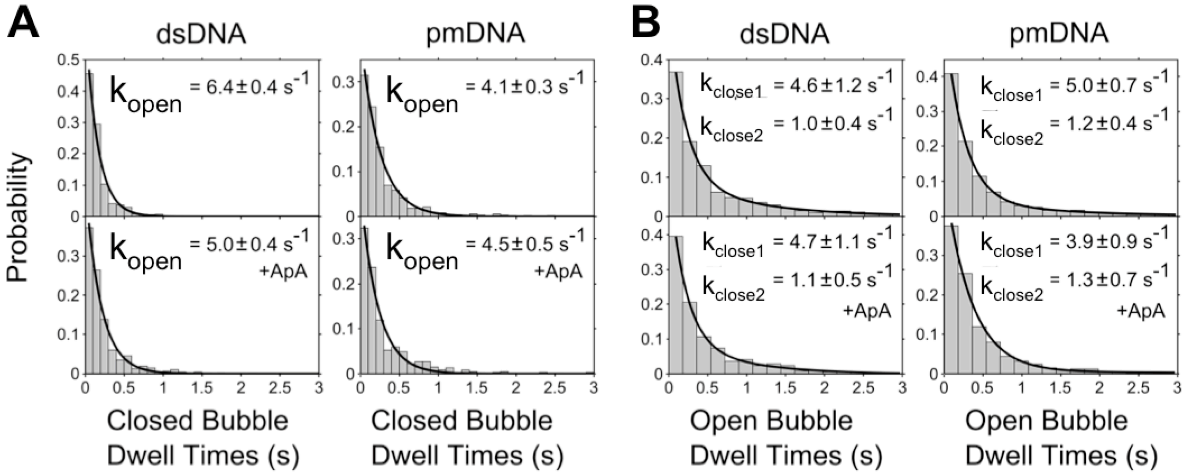

Figure S6. The presence of ApA dinucleotide has little effect on transcription-bubble dynamics. (A) Stacked histograms of closed conformation dwell times for dsDNA and pmDNA. The data are fit to single-exponential functions. The bubble-opening rates are shown inset. (B) Stacked histograms of the open bubble conformation dwell times for dsDNA and pmDNA. The data are fit to double-exponential functions. The bubble closing rates are shown inset. The wild-type data in the top panels is identical to the one in the main text, and is present here again for comparison purposes. The bottom panel shows data for wt holoenzyme in the presence of 2 mM ApA.
